# Some Generating Functions for *q*-Polynomials

**DOI:** 10.3390/sym10120758

**Published:** 2018

**Authors:** Howard S. Cohl, Roberto S. Costas-Santos, Tanay V. Wakhare

**Affiliations:** 1 Applied and Computational Mathematics Division, National Institute of Standards and Technology, Mission Viejo, CA 92694, USA; 2 Departamento de Física y Matemáticas, Facultad de Ciencias, Universidad de Alcalá, Alcalá de Henares, 28871 Madrid, Spain; 3 Department of Mathematics, University of Maryland, College Park, MD 20742, USA

**Keywords:** basic hypergeometric functions, generating functions, *q*-polynomials, 33D45, 33C20

## Abstract

Demonstrating the striking symmetry between calculus and *q*-calculus, we obtain *q*-analogues of the Bateman, Pasternack, Sylvester, and Cesàro polynomials. Using these, we also obtain *q*-analogues for some of their generating functions. Our *q*-generating functions are given in terms of the basic hypergeometric series _4_*ϕ*_5_, _5_*ϕ*_5_, _4_*ϕ*_3_, _3_*ϕ*_2_, _2_*ϕ*_1_, and *q*-Pochhammer symbols. Starting with our *q*-generating functions, we are also able to find some new classical generating functions for the Pasternack and Bateman polynomials.

## 1. Introduction

We will adopt the following notations for sets: ℕ:={1,2,3,…}, ${\mathbb{N}}_{0}:=\{0,1,2,\ldots \}$, and ℂ is the set of complex numbers. We also adopt the conventions that an empty sum vanishes and the empty product is unity.

The generalized hypergeometric series _*r*_*F*_*s*_ is given by [1] (1.4.1)

$${}_{r}F_{s}\left(\begin{array}{l} a_{1},\ldots ,a_{r}\\ b_{1},\ldots ,b_{s} \end{array};z\right)=\sum_{n=0}^{\infty }\frac{{\left(a_{1}\right)}_{n}\;\ldots \;{\left(a_{r}\right)}_{n}}{{\left(b_{1}\right)}_{n}\;\ldots \;{\left(b_{s}\right)}_{n}}\frac{z^{n}}{n!},$$

where |*z*| < 1, the Pochhammer symbol (rising factorial) is defined by (*a*)_*n*_ := (*a*)(*a* + 1) · · · (*a* + *n* − 1), and the (*b*_*i*_) are such that these denominator factors never vanish. A *q*-analogue of the hypergeometric series _*r*_*F*_*s*_ is the basic hypergeometric series [2]

(1) $${}_{r}\phi_{s}\left(\begin{array}{l} a_{1},a_{2},\ldots ,a_{r}\\ b_{1},b_{2},\ldots ,b_{s} \end{array}|q,z\right)=\sum_{n=0}^{\infty }\frac{{\left(a_{1};\;q\right)}_{n}{\left(a_{2};\;q\right)}_{n}\;\ldots \;{\left(a_{r};\;q\right)}_{n}}{{\left(b_{1};\;q\right)}_{n}{\left(b_{2};\;q\right)}_{n}\;\ldots \;{\left(b_{s};\;q\right)}_{n}}{\left[{\left(-1\right)}^{n}q^{\frac{n(n-1)}{2}}\right]}^{1+s-r}\frac{z^{n}}{{\left(q;\;q\right)}_{n}},$$

where *q* ≠ 0 when *r* > *s* + 1. We refer to [2] for convergence properties of the series, however note that (*b*_*i*_) is such that the denominator factors never vanish. This occurs when *b*_*i*_ = *q*^−*m*^ for some $m\in {\mathbb{N}}_{0}$. Further note the important limit [1] (p. 15)

(2) limq↑1ϕrs(qa1,qa2,…,qarqb1,qb2,…,qbs∣q,(q−1)1+s−rz)=Frs(a1,a2,…,arb1,b2,…,bs;z).

The *q*-Pochhammer symbol with non-negative integer subscript is defined by

$${\left(a;q\right)}_{0}=1,\;{\left(a;q\right)}_{n}=(1-a)(1-q)\;\ldots \;\left(1-aq^{n-1}\right),\;n\in \mathbb{N}.$$

Some useful properties of the *q*-Pochhammer symbols that we will take advantage of with $n\in {\mathbb{N}}_{0}$ include [1] (1.8.18)

(3) $${\left(q^{-n};\;q\right)}_{k}=\frac{{\left(q;\;q\right)}_{n}}{{\left(q;\;q\right)}_{n-k}}{\left(-1\right)}^{k}q^{\frac{k(k-1)}{2}-nk},\;k=0,1,\ldots ,n$$

and [1] (1.8.21–22)

(4) $${\left(a;\;q\right)}_{2n}={\left(a^{\frac{1}{2}};\;q\right)}_{n}{\left(-a^{\frac{1}{2}};\;q\right)}_{n}{\left({\left(aq\right)}^{\frac{1}{2}};\;q\right)}_{n}{\left(-{\left(aq\right)}^{\frac{1}{2}};\;q\right)}_{n}.$$

Furthermore, one has [1] (p. 12)

$${\left(a;\;q\right)}_{\infty }:=\prod_{k=1}^{\infty }\left(1-aq^{k-1}\right),$$

and a generalization of the *q*-Pochhammer symbol for arbitrary λ∈ℂ is given by [1] (1.8.9)

(5) $${\left(a;\;q\right)}_{\lambda }:=\frac{{\left(a;\;q\right)}_{\infty }}{{\left(aq^{\lambda };\;q\right)}_{\infty }},\;|q|<1,$$

where the principal value of *q*^*λ*^ is taken. Note that, from (5), it follows that, for *a*, *α*, β∈ℂ,

$${\left(a;\;q\right)}_{\alpha +\beta }={\left(a;\;q\right)}_{\alpha }{\left(aq^{\alpha };\;q\right)}_{\beta }.$$

We need to define some other *q*-analogues, such as the *q*-analogue of a (real) number [*a*]_*q*_, and the *q*-factorial [*n*]_*q*_!. For the *q*-number one has [1] (1.8.1)

$${\left[a\right]}_{q}:=\frac{1\;-\;q^{a}}{1\;-\;q},$$

where *q* ≠ 0, *q* ≠ 1, and the *q*-factorial [2] (1.2.44)

$${\text{[0]}}_{q}!=1,\;{\text{[}n\text{]}}_{q}\text{!}=\prod_{k=1}^{n}{\left[k\right]}_{q},\;\;n\in \mathbb{N}.$$

We also will need a *q*-analogue of the *binomial theorem* [2] (1.3.2)

(6) ϕ10(a−∣q,z)=∑k=0∞(a;q)k(q;q)kzk=(az;q)∞(z;q)∞,|z|<1,|q|<1.

The *q*-binomial coefficient is defined for *a*, b∈ℂ, [2] (I.40)

$${\left[\begin{array}{l} a\\ b \end{array}\right]}_{q}:=\frac{{\left(q^{b+1};\;q\right)}_{\infty }{\left(q^{a-b+1};\;q\right)}_{\infty }}{{\left(q;\;q\right)}_{\infty }{\left(q^{a+1};\;q\right)}_{\infty }},$$

which specializes if $n\in {\mathbb{N}}_{0}$, *k* = 0, 1, . . . , *n*, to [2] (I.39)

$${\left[\begin{array}{l} n\\ k \end{array}\right]}_{q}=\frac{{\left(q;\;q\right)}_{n}}{{\left(q;\;q\right)}_{k}{\left(q;\;q\right)}_{n-k}}.$$

### Mathematical and Physical Applications

H. Bateman introduced in [3], the hypergeometric polynomial which we refer to in this paper as the Bateman polynomials. The goal of his research in the direction of these polynomials was in trying to better understand some radiation and conduction problems in which one requires the inverse of the Laplace transform. Our generalized *q*-generating functions for the Bateman and Pasternack (generalized Bateman) polynomials may very well be useful to the study of *q*-analogues of these radiation and conduction problems. Sylvester (1879) [4] investigated his polynomials and showed that the numbers $\varphi_{n}\left(\frac{1}{4}\right)$ can be used for the computation of the numbers of different terms in the determinant of a skew-symmetric matrix of degree 2*n*. Similarly, $\varphi_{n}\left(\frac{1}{8}\right)$ is significant for the computation of the number of different terms in a determinant of degree 4*n*, which is skew-symmetric with respect to both diagonals (see [5] (pp. 255–256)). Cesàro polynomials $g_{n}^{\left(s\right)}(z)$ are in fact the *s*th mean of the first *n* partial sums of 1 + *x* + *x*^2^ + . . . (see [6] (p. 185)). Our generalizations should apply in *q*-generalizations of all of these problems, such as for the *q*-Laplace transform and for *q*-Bernstein polynomials.

## 2. The Bateman, Sylvester, Pasternack, and Cesàro Polynomials

H. Bateman introduced in [3], the generalized hypergeometric polynomial:

𝒵n(z)=F22(−n,n+11,1|z).

By using [7] (Theorem 48), we obtain the generating function

∑n=0∞𝒵n(z)tn=11−tF11(121|−4zt(1−t)2).

Using the above information, the Bateman polynomials are defined as [8] (p. 25)

ℬn(z)=F32(−n,n+1,z+121,1|1).

He also obtained the generating functions:

∑n=0∞ℬn(z)tn=11−tF21(12,z+121|−4t(1−t)2),

∑n=0∞(ℬn(z−2)−ℬn(z))tn=2t(1−t)3F21(32,z+122|−4t(1−t)2).

### Lemma 1.

Let t∈ℂ. Then the following relation holds:

(7) $$\sum_{n=0}^{\infty }{ℬ}_{m}(-2n-1)\frac{t^{n}}{n!}=e^{t}{𝒵}_{m}(-t).$$

The Bateman polynomial ${ℬ}_{n}$ was generalized by Pasternack in [9]. He defines the polynomial ${ℬ}_{n}^{m}$ as

ℬnm(z)=F32(−n,n+1,z+m+121,m+1|1)

for m∈ℂ\{−1}. These polynomials reduce to the Bateman polynomials when *m* = 0. Further information regarding such polynomials and their connection with (classical) orthogonal polynomials can be found in [10]. Indeed, we can write the Pasternack polynomials in terms of the continuous Hahn polynomials as follows [10] (p. 893):

$${ℬ}_{n}^{m}(z)=\frac{1}{i^{n}{\left(m\;+\;1\right)}_{n}}p_{n}\left(\frac{-iz}{2};\;\frac{1\;+\;m}{2},\frac{1\;-\;m}{2},\frac{1\;-\;m}{2},\frac{1\;+\;m}{2}\right).$$

We also consider the Sylvester polynomials, defined as (see [6] (p. 185))

φn(z)=znn!F20(−n,z−|−1z).

Notice that we also can write the Sylvester polynomials in terms of (classical) orthogonal polynomials [1] (p. 48)

$$\varphi_{n}(z)={\left(-1\right)}^{n}L_{n}^{\left(-z-n\right)}(x)=\frac{z^{n}}{n!}C_{n}(-z;\;z).$$

Here $L_{n}^{\left(\alpha \right)}$ and *C*_*n*_ represent the Laguerre and Charlier polynomials. It is also known that the Sylvester polynomials satisfy the generating functions

$$\sum_{n=0}^{\infty }\varphi_{n}(z)t^{n}=\frac{e^{zt}}{{\left(1\;-\;t\right)}^{z}},$$

∑n=0∞(λ)nφn(z)tn=1(1−zt)λF20(λ,z−|t1−zt).

The Cesàro polynomials are defined as [6] (p. 449)

gn(s)(z)=(1+s)nn!F21(−n,1−s−n|z).

Observe that this family can be written in terms of Jacobi polynomials [6] (p. 449) as

(8) $$g_{n}^{\left(s\right)}(z)=P_{n}^{\left(s+1,-s-n-1\right)}(2z-1).$$

Furthermore, they satisfy the generating functions: [11] (4.2)

(9) $$\sum_{n=0}^{\infty }g_{n}^{\left(s\right)}(z)t^{n}={\left(1-t\right)}^{-s-1}{\left(1-zt\right)}^{-1},$$

(10) $$\sum_{n=0}^{\infty }\left(\begin{array}{c} n+\ell \\ \ell \end{array}\right)g_{n+\ell }^{\left(s\right)}(z)t^{n}={\left(1-t\right)}^{-s-1-\ell }{\left(1-zt\right)}^{-1}g_{\ell }^{\left(s\right)}\left(\frac{z(1\;-\;t)}{1\;-\;zt}\right).$$

The aim of this paper is to obtain the *q*-analogue of all these families of polynomials as well as the *q*-analogues of the generating functions stated above. The structure of this paper is as follows. In Section 2, we give some preliminaries on *q*-calculus and we define some *q*-analogues of the Bateman, Sylvester, Pasternack, and Cesàro polynomials. In Section 3, we state and prove most (see Remark 1 below) of the *q*-analogues of the generating functions associated with the *q*-Bateman, *q*-Sylvester, *q*-Pasternack, and *q*-Cesàro polynomials.

## 3. The *q*-Analogues of the Bateman, Sylvester, Pasternack, and Cesàro Polynomials

Taking into account the *q*-definitions in Section 1 and the polynomials introduced therein, we define the *q*-Bateman polynomial as

𝒵n(z;q)=ϕ22(q−n,qn+1q,q|q,qnz),

ℬn(z;q)=ϕ32(q−n,qn+1,q1+z2q,q|q,qn),

define the *q*-Pasternack polynomial as

ℬnm(z;q)=ϕ32(q−n,qn+1,q1+z+m2q,qm+1|q,qn),

define the *q*-Sylvester polynomial as

φn(z;q)=zn(q;q)nϕ20(q−n,qz−|q,qnz−1),

and define the *q*-Cesàro polynomial as

gn(s)(z;q)=(qs+1;q)n(q;q)nϕ21(q−n,qq−s−n|q,z).

### Lemma 2.

The q-Cesàro polynomial can be written as

(11) $$g_{n}^{\left(s\right)}(z;\;q)=\sum_{k=0}^{n}{\left[\begin{array}{c} k+s\\ s \end{array}\right]}_{q}{\left(zq^{s}\right)}^{n-k}.$$

## 4. The Generating Functions

### Theorem 1.

*Let q*, *t*, z∈ℂ, |*q*| < 1*,* |*t*| < 1*,* |*z*| < 1. *Then the q-Bateman polynomials satisfy the following generating functions:*

(12) ∑n=0∞ℬn(z;q)tn=11−tϕ55(−q,q12,−q12,qz+12,0q,qt12,−qt12,(qt)12,−(qt)12|q,t),

(13) ∑n=0∞𝒵n(z;q)tn=11−tϕ45(−q,q12,−q12,0q,qt12,−qt12,(q)12,−(qt)12|q,zt),

(14) ∑n=0∞(ℬn(z−2;q)−ℬn(z;q))tn=(1+q)qz−12t(t;q)3ϕ55(−q2,q32,−q32,qz+12,0q2,q2t12,−q2t12,(q3t)12,−(q3t)12|q,qt).

### Proof.

Let us start by proving Identity (12). One has

$$\sum_{n=0}^{\infty }{ℬ}_{n}(z;\;q)t^{n}=\sum_{n=0}^{\infty }\sum_{k=0}^{n}\frac{{\left(q^{-n};\;q\right)}_{k}{\left(q^{n+1};\;q\right)}_{k}{\left(q^{\frac{z+1}{2}};\;q\right)}_{k}}{{\left(q;\;q\right)}_{k}{\left(q;\;q\right)}_{k}{\left(q;\;q\right)}_{k}}{\left(q^{k}t\right)}^{n}.$$

Using (3), we obtain

$$\sum_{n=0}^{\infty }{ℬ}_{n}(z;\;q)t^{n}=\sum_{n=0}^{\infty }\sum_{k=0}^{n}\frac{{\left(q;\;q\right)}_{n+k}{\left(q^{\frac{z+1}{2}};\;q\right)}_{k}}{{\left(q;\;q\right)}_{n-k}{\left(q;\;q\right)}_{k}{\left(q;\;q\right)}_{k}{\left(q;\;q\right)}_{k}}{\left(-1\right)}^{k}q^{\frac{k(k-1)}{2}}t^{n}.$$

Next, we rearrange the double summation and set *n* ↦ *n* + *k*, obtaining

$$\begin{array}{l} \sum_{n=0}^{\infty }{ℬ}_{n}(z;\;q)t^{n}=\sum_{k=0}^{\infty }\sum_{n=0}^{\infty }\frac{{\left(q;\;q\right)}_{n+2k}{\left(q^{\frac{z+1}{2}};\;q\right)}_{k}}{{\left(q;\;q\right)}_{n}{\left(q;\;q\right)}_{k}{\left(q;\;q\right)}_{k}{\left(q;\;q\right)}_{k}}{\left(-1\right)}^{k}q^{\frac{k(k-1)}{2}}t^{n+k}\\ \;=\sum_{k=0}^{\infty }\frac{{\left(q;\;q\right)}_{2k}{\left(q^{\frac{z+1}{2}};\;q\right)}_{k}}{{\left(q;\;q\right)}_{k}{\left(q;\;q\right)}_{k}{\left(q;\;q\right)}_{k}}{\left(-t\right)}^{k}q^{\frac{k(k-1)}{2}}\sum_{n=0}^{\infty }\frac{{\left(q^{2k+1};\;q\right)}_{n}}{{\left(q;\;q\right)}_{n}}t^{n}. \end{array}$$

By using (4) and the *q*-analogue of the binomial theorem (6), we obtain

$$\begin{array}{l} =\sum_{k=0}^{\infty }\frac{{\left(-q;\;q\right)}_{k}{\left(q^{\frac{1}{2}};\;q\right)}_{k}{\left(-q^{\frac{1}{2}};\;q\right)}_{k}{\left(q^{\frac{z+1}{2}};\;q\right)}_{k}}{{\left(q;\;q\right)}_{k}{\left(q;\;q\right)}_{k}}{\left(-t\right)}^{k}q^{\frac{k(k-1)}{2}}\frac{{\left(q^{2k+1}t;\;q\right)}_{\infty }}{{\left(t;\;q\right)}_{\infty }}\\ =\sum_{k=0}^{\infty }\frac{{\left(-q;\;q\right)}_{k}{\left(q^{\frac{1}{2}};\;q\right)}_{k}{\left(-q^{\frac{1}{2}};\;q\right)}_{k}{\left(q^{\frac{z+1}{2}};\;q\right)}_{k}}{{\left(t;\;q\right)}_{2k+1}{\left(q;\;q\right)}_{k}{\left(q;\;q\right)}_{k}}{\left(-t\right)}^{k}q^{\frac{k(k-1)}{2}}\\ =\frac{1}{1\;-\;t}\sum_{k=0}^{\infty }\frac{{\left(-q;\;q\right)}_{k}{\left(q^{\frac{1}{2}};\;q\right)}_{k}{\left(-q^{\frac{1}{2}};\;q\right)}_{k}{\left(q^{\frac{z+1}{2}};\;q\right)}_{k}}{{\left(qt;\;q\right)}_{2k}{\left(q;\;q\right)}_{k}{\left(q;\;q\right)}_{k}}{\left(-t\right)}^{k}q^{\frac{k(k-1)}{2}}\\ =\frac{1}{1\;-\;t}\sum_{k=0}^{\infty }\frac{{\left(-q;\;q\right)}_{k}{\left(q^{\frac{1}{2}};\;q\right)}_{k}{\left(-q^{\frac{1}{2}};\;q\right)}_{k}{\left(q^{\frac{z+1}{2}};\;q\right)}_{k}}{{\left(q;\;q\right)}_{k}{\left({\left(qt\right)}^{\frac{1}{2}};\;q\right)}_{k}{\left(-{\left(qt\right)}^{\frac{1}{2}};\;q\right)}_{k}{\left(qt^{\frac{1}{2}};\;q\right)}_{k}{\left(-qt^{\frac{1}{2}};\;q\right)}_{k}}{\left(-1\right)}^{k}q^{\frac{k(k-1)}{2}}\frac{t^{k}}{{\left(q;\;q\right)}_{k}}. \end{array}$$

Hence, the identity follows. In order to prove Identity (14), one has

$$\begin{array}{l} \sum_{n=0}^{\infty }\left({ℬ}_{n}(z-2;\;q)-{ℬ}_{n}(z;\;q)\right)t^{n}=\sum_{n=0}^{\infty }\sum_{k=0}^{n}\frac{{\left(q^{-n};\;q\right)}_{k}{\left(q^{n+1};\;q\right)}_{k}}{{\left(q;\;q\right)}_{k}{\left(q;\;q\right)}_{k}{\left(q;\;q\right)}_{k}}q^{nk}\left({\left(q^{\frac{z-1}{2}};\;q\right)}_{k}-{\left(q^{\frac{z+1}{2}};\;q\right)}_{k}\right)t^{n}\\ \;=\sum_{n=0}^{\infty }\sum_{k=0}^{n}\frac{{\left(q^{-n};\;q\right)}_{k}{\left(q^{n+1};\;q\right)}_{k}}{{\left(q;\;q\right)}_{k}{\left(q;\;q\right)}_{k}{\left(q;\;q\right)}_{k}}q^{nk}{\left(q^{\frac{z+1}{2}};\;q\right)}_{k-1}\left(1-q^{k}\right)\left(-q^{\frac{z-1}{2}}\right)t^{n}\\ \;=\sum_{n=0}^{\infty }\sum_{k=0}^{n}\frac{{\left(q;\;q\right)}_{n+k}{\left(q^{\frac{z+1}{2}};\;q\right)}_{k-1}{\left(-1\right)}^{k-1}q^{\frac{z-1}{2}}q^{\frac{k(k-1)}{2}}}{{\left(q;\;q\right)}_{n-k}{\left(q;\;q\right)}_{k-1}{\left(q;\;q\right)}_{k}{\left(q;\;q\right)}_{k}}t^{n}. \end{array}$$

Taking into account that the above expression vanishes at *k* = 0, we set *k* ↦ *k* + 1, rearrange the double sum, and set *n* ↦ *n* + *k*, yielding

$$=\sum_{k=0}^{\infty }\sum_{n=0}^{\infty }\frac{{\left(q;\;q\right)}_{n+2k+1}{\left(q^{\frac{z+1}{2}};\;q\right)}_{k}{\left(-1\right)}^{k}q^{\frac{z-1}{2}}q^{\frac{k(k+1)}{2}}}{{\left(q;\;q\right)}_{n-1}{\left(q;\;q\right)}_{k}{\left(q;\;q\right)}_{k+1}{\left(q;\;q\right)}_{k+1}}t^{n+k}.$$

Here, again, the series vanishes at *n* = 0, so we set *n* ↦ *n* + 1. Applying some basic identities of *q*-Pochhammer symbols, we obtain

$$\begin{array}{l} =(1+q)q^{\frac{z-1}{2}}t\sum_{k=0}^{\infty }\sum_{n=0}^{\infty }\frac{{\left(q^{3};\;q\right)}_{n+2k}{\left(q^{\frac{z+1}{2}};\;q\right)}_{k}{\left(-1\right)}^{k}q^{\frac{k(k-1)}{2}}}{{\left(q;\;q\right)}_{n}{\left(q;\;q\right)}_{k}{\left(q^{2};\;q\right)}_{k}{\left(q^{2};\;q\right)}_{k}}{\left(qt\right)}^{k}t^{n}\\ =(1+q)q^{\frac{z-1}{2}}t\sum_{k=0}^{\infty }\frac{{\left(q^{3};\;q\right)}_{2k}{\left(q^{\frac{z+1}{2}};\;q\right)}_{k}}{{\left(q;\;q\right)}_{k}{\left(q^{2};\;q\right)}_{k}{\left(q^{2};\;q\right)}_{k}}{\left(-1\right)}^{k}q^{\frac{k(k-1)}{2}}{\left(qt\right)}^{k}\sum_{n=0}^{\infty }\frac{{\left(q^{3+2k};\;q\right)}_{n}}{{\left(q;\;q\right)}_{n}}t^{n}. \end{array}$$

Applying again the *q*-analogue of the binomial theorem (6) and simplifying, we obtain

$$\begin{array}{l} =(1+q)q^{\frac{z-1}{2}}t\sum_{k=0}^{\infty }\frac{{\left(q^{3};\;q\right)}_{2k}{\left(q^{\frac{z+1}{2}};\;q\right)}_{k}}{{\left(q;\;q\right)}_{k}{\left(q^{2};\;q\right)}_{k}{\left(q^{2};\;q\right)}_{k}}{\left(-1\right)}^{k}q^{\frac{k(k-1)}{2}}{\left(qt\right)}^{k}\frac{1}{{\left(t;\;q\right)}_{3+2k}}\\ =(1+q)q^{\frac{z-1}{2}}t\sum_{k=0}^{\infty }\frac{{\left(q^{\frac{3}{2}};\;q\right)}_{k}{\left(-q^{\frac{3}{2}};\;q\right)}_{k}{\left(-q^{2};\;q\right)}_{k}{\left(q^{\frac{z+1}{2}};\;q\right)}_{k}}{{\left(q;\;q\right)}_{k}{\left(q^{2};\;q\right)}_{k}}{\left(-1\right)}^{k}q^{\frac{k(k-1)}{2}}{\left(qt\right)}^{k}\frac{1}{{\left(t;\;q\right)}_{3+2k}}\\ =\frac{\left(1\;+\;q\right)}{{\left(t;\;q\right)}_{3}}q^{\frac{z-1}{2}}t\sum_{k=0}^{\infty }\frac{{\left(q^{\frac{3}{2}};\;q\right)}_{k}{\left(-q^{\frac{3}{2}};\;q\right)}_{k}{\left(-q^{2};\;q\right)}_{k}{\left(q^{\frac{z+1}{2}};\;q\right)}_{k}{\left(-1\right)}^{k}q^{\frac{k(k-1)}{2}}}{{\left(q;\;q\right)}_{k}{\left(q^{2};\;q\right)}_{k}{\left({\left(tq^{3}\right)}^{\frac{1}{2}};\;q\right)}_{k}{\left(-{\left(tq^{3}\right)}^{\frac{1}{2}};\;q\right)}_{k}{\left(q^{2}t^{\frac{1}{2}};\;q\right)}_{k}{\left(-q^{2}t^{\frac{1}{2}};\;q\right)}_{k}}{\left(qt\right)}^{k}. \end{array}$$

Therefore, the identity follows. Let us prove the generating function Equation (14). We have

$$\begin{array}{l} \sum_{n=0}^{\infty }{𝒵}_{n}(z;\;q)t^{n}=\sum_{n=0}^{\infty }\sum_{k=0}^{n}\frac{{\left(q^{-n};\;q\right)}_{k}{\left(q^{n+1};\;q\right)}_{k}{\left(-1\right)}^{k}q^{\frac{k(k-1)}{2}}}{{\left(q;\;q\right)}_{k}{\left(q;\;q\right)}_{k}{\left(q;\;q\right)}_{k}}z^{k}q^{nk}t^{n}\\ \;=\sum_{n=0}^{\infty }\sum_{k=0}^{n}\frac{{\left(q;\;q\right)}_{n+k}q^{k(k-1)}}{{\left(q;\;q\right)}_{n-k}{\left(q;\;q\right)}_{k}{\left(q;\;q\right)}_{k}{\left(q;\;q\right)}_{k}}z^{k}t^{n}. \end{array}$$

As in the previous identities, we rearrange the double sums and set *n* ↦ *n* + *k*, obtaining

$$\begin{array}{l} =\sum_{k=0}^{\infty }\sum_{n=0}^{\infty }\frac{{\left(q;\;q\right)}_{n+2k}q^{k(k-1)}}{{\left(q;\;q\right)}_{n}{\left(q;\;q\right)}_{k}{\left(q;\;q\right)}_{k}{\left(q;\;q\right)}_{k}}z^{k}t^{n+k}=\sum_{k=0}^{\infty }\frac{{\left(q;\;q\right)}_{2k}q^{k(k-1)}}{{\left(q;\;q\right)}_{k}{\left(q;\;q\right)}_{k}{\left(q;\;q\right)}_{k}}{\left(zt\right)}^{k}\sum_{n=0}^{\infty }\frac{{\left(q^{2k+1};\;q\right)}_{n}}{{\left(q;\;q\right)}_{n}}t^{n}\\ =\sum_{k=0}^{\infty }\frac{{\left(q;\;q\right)}_{2k}q^{k(k-1)}}{{\left(q;\;q\right)}_{k}{\left(q;\;q\right)}_{k}{\left(q;\;q\right)}_{k}}{\left(zt\right)}^{k}\frac{1}{{\left(t;\;q\right)}_{2k+1}}=\frac{1}{1\;-\;t}\sum_{k=0}^{\infty }\frac{{\left(-q;\;q\right)}_{k}\left(q^{\frac{1}{2}};\;q\right)\left(-q^{\frac{1}{2}};\;q\right)q^{k(k-1)}}{{\left(q;\;q\right)}_{k}{\left(q;\;q\right)}_{k}{\left(tq;\;q\right)}_{2k}}{\left(zt\right)}^{k}\\ =\frac{1}{1\;-\;t}\sum_{k=0}^{\infty }\frac{{\left(-q;\;q\right)}_{k}\left(q^{\frac{1}{2}};\;q\right)\left(-q^{\frac{1}{2}};\;q\right)q^{k(k-1)}}{{\left(q;\;q\right)}_{k}{\left(q;\;q\right)}_{k}{\left({\left(tq\right)}^{\frac{1}{2}};\;q\right)}_{k}{\left(-{\left(tq\right)}^{\frac{1}{2}};\;q\right)}_{k}{\left(qt^{\frac{1}{2}};\;q\right)}_{k}{\left(-qt^{\frac{1}{2}};\;q\right)}_{k}}{\left(zt\right)}^{k}. \end{array}$$

Hence, the identity follows since ${\left({\left(-1\right)}^{k}q^{\frac{k(k-1)}{2}}\right)}^{2}=q^{k(k-1)}$. Note that, in order for the generating functions to converge, one must require |*t*| < 1. Furthermore, one must also have that the denominator parameters of the basic hypergeometric series must not be equal to a factor of *q*^−*m*^ for some $m\in {\mathbb{N}}_{0}$. This requires that |*t*| < |*q*|^−1^, which is greater than unity since |*q*| < 1, so |*t*| < 1 suffices. Since |*t*| < 1, then |*z*| < 1 as well. This completes the proof. □

### Theorem 2.

*Let j*, $m\in {\mathbb{N}}_{0}$, *q*, *t*, λ∈ℂ, |*q*| < 1, |*t*| < 1. *Then the following identities hold:*

(15) ∑n=0∞ℬmj(−2n−j−1;q)(qλ;q)n(q;q)ntn=1(t;q)λϕ43(q−m,qm+1,qλ,0q,qj+1,qt−1|q,qm),

(16) ∑n=0∞ℬmj(−2n−j−1;q)tn=11−tϕ32(q−m,qm+1,0qj+1,qt−1∣q,qm),

(17) ∑n=0∞ℬm(−2n−1;q)tn(q;q)n=1(t;q)∞ϕ43(q−m,qm+1,0,0q,q,qt−1|q,qm),

where the principal value of q^λ^ is taken.

### Proof.

Let us prove (15). Setting *z* = −2*n* − 1 − *j* in the *q*-Pasternack polynomial, and using their basic hypergeometric expansion, we have

∑n=0∞ℬmj(−2n−1−j;q)(qλ;q)n(q;q)ntn=∑n=0∞∑k=0n(q−m;q)k(qm+1;q)k(q−n;q)k(q;q)k(q;q)k(qj+1;q)kqnktn(qλ;q)n(q;q)n=∑n=0∞(qλ;q)ntn(q;q)n∑k=0min(m,n)(q−m;q)k(qm+1;q)k(q−n;q)kqmk(q;q)k(q;q)k(qj+1;q)k=∑k=0m(q−m;q)k(qm+1;q)k(qλ;q)k(tqm)k(q;q)k(q;q)k(q;q)k(qj+1;q)k∑n=0∞(q−n−k;q)k(qλ+k;q)ntn(qk+1;q)n=∑k=0m(q−m;q)k(qm+1;q)k(qλ;q)k(−t)kqmk+(2k)−k2(q;q)k(q;q)k(qj+1;q)k∑n=0∞(qλ+k;q)ntnq−nk(q;q)n=∑k=0m(q−m;q)k(qm+1;q)k(qλ;q)k(−t)kqmk+(2k)−k2(q;q)k(q;q)k(qj+1;q)kϕ10(qλ+k−|q,tq−k)=1(t;q)λϕ43(q−m,qm+1,qλ,0q,qj+1,qt−1∣q,t),

where we have used (5), (6) [1] (1.8.6), which completes the proof of (15). Observe that, if we set *λ* = 1 in (15), we obtain (16). Since |*q*| < 1, taking the limit *λ* → ∞ yields (17). Note that, in order for the generating functions to converge, one must require |*t*| < 1. This completes the proof. □

Setting *t* ↦ *t*(1 − *q*) and taking the *q* ↑ 1 limit of (17) produces Lemma 1 since

$$\underset{q↑1}{\lim}\frac{{\left(1\;-\;q\right)}^{n}}{{\left(q;\;q\right)}_{n}}=\frac{1}{n{!}^{\prime }},\;\underset{q↑1}{\lim}\frac{1}{{\left(t(1\;-\;q);\;q\right)}_{\infty }}=e^{t},$$

limq↑1ϕ43(q−m,qm+1,0,0q,q,q(1−q)t∣q,qm)=F22(−m,m+11,1|−t),

which follows easily by expanding the denominator factor involving *t* and using (2).

In fact, we are now able to obtain new classical generating functions for the Pasternack and Bateman polynomials by taking the *q* ↑ 1 limit in (15), (16).

### Corollary 1.

*Let j*, $m\in {\mathbb{N}}_{0}$, *t*, λ∈ℂ. *Then the following identities hold:*

(18) ∑n=0∞ℬmj(−2n−1−j)(λ)nn!tn=1(1−t)λF32(−m,m+1,λ1,j+1;−t1−t),

(19) ∑n=0∞ℬm(−2n−1)(λ)nn!tn=1(1−t)λF32(−m,m+1,λ1,1;−t1−t),

(20) ∑n=0∞ℬmj(−2n−1−j)tn=11−tF21(−m,m+1j+1;−t1−t),

(21) $$\sum_{n=0}^{\infty }{ℬ}_{m}(-2n-1)t^{n}=\frac{1}{1-t}P_{m}\left(\frac{1\;+\;t}{1\;-\;t}\right),$$

*where P*_*m*_(*x*) *is the Legendre polynomial [1] (Section 9.8.3).*

### Proof.

In (15), take the *q* ↑ 1 limit. Note that

(t;q)λ=(t;q)∞(tqλ;q)∞=ϕ10(q−λ−|q,tqλ).

Thus, by using (2),

$$\underset{q↑1}{\lim}{\left(t;\;q\right)}_{\lambda }{=}_{1}F_{0}(\begin{array}{c} -\lambda \\ - \end{array}|t)={\left(1-t\right)}^{\lambda }.$$

Hence by expanding the denominator factor involving *t* using (2) in (15) produces (18). Setting *j* = 0 and *λ* = 1 in (18), produces (19), (20), respectively. Setting *j* = 0 in (20) produces (21), by noting [1] (9.8.62). □

### Theorem 3.

*Let* $m\in {\mathbb{N}}_{0}$, *q*, *t*, z∈ℂ, |*q*| < 1, |*t*| < 1. *Then the q-Pasternack polynomials satisfy the following generating function:*

(22) ∑n=0∞ℬnm(z;q)tn=11−tϕ55(−q,q12,−q12,qz+m+12,0qm+1,qt12,−qt12,(qt)12,−(qt)12|q,t).

### Proof.

Taking into account the expression of the basic hypergeometric series for these polynomials, we have

$$\begin{array}{l} \sum_{n=0}^{\infty }{ℬ}_{n}^{m}(z;\;q)t^{n}=\sum_{n=0}^{\infty }\sum_{k=0}^{n}\frac{{\left(q^{-n};\;q\right)}_{k}{\left(q^{n+1};\;q\right)}_{k}{\left(q^{\frac{z+m+1}{2}};\;q\right)}_{k}}{{\left(q;\;q\right)}_{k}{\left(q;\;q\right)}_{k}{\left(q^{m+1};\;q\right)}_{k}}{\left(q^{k}t\right)}^{n}\\ \;=\sum_{n=0}^{\infty }\sum_{k=0}^{n}\frac{{\left(q;\;q\right)}_{n+k}{\left(q^{\frac{z+m+1}{2}};\;q\right)}_{k}}{{\left(q;\;q\right)}_{n-k}{\left(q;\;q\right)}_{k}{\left(q;\;q\right)}_{k}{\left(q^{m+1};\;q\right)}_{k}}{\left(-1\right)}^{k}q^{\frac{k(k-1)}{2}}t^{n}\\ \;=\sum_{k=0}^{\infty }\sum_{n=0}^{\infty }\frac{{\left(q;\;q\right)}_{n+2k}{\left(q^{\frac{z+m+1}{2}};\;q\right)}_{k}}{{\left(q;\;q\right)}_{n}{\left(q;\;q\right)}_{k}{\left(q;\;q\right)}_{k}{\left(q^{m+1};\;q\right)}_{k}}{\left(-1\right)}^{k}q^{\frac{k(k-1)}{2}}t^{n+k}\\ \;=\sum_{k=0}^{\infty }\frac{{\left(q;\;q\right)}_{2k}{\left(q^{\frac{z+m+1}{2}};\;q\right)}_{k}}{{\left(q;\;q\right)}_{k}{\left(q;\;q\right)}_{k}{\left(q^{m+1};\;q\right)}_{k}}{\left(-1\right)}^{k}q^{\frac{k(k-1)}{2}}t^{k}\frac{{\left(q^{2k+1}t;\;q\right)}_{\infty }}{{\left(t;\;q\right)}_{\infty }}\\ \;=\frac{1}{1\;-\;t}\sum_{k=0}^{\infty }\frac{{\left(q;\;q\right)}_{2k}{\left(q^{\frac{z+m+1}{2}};\;q\right)}_{k}}{{\left(q;\;q\right)}_{k}{\left(q;\;q\right)}_{k}{\left(q^{m+1};\;q\right)}_{k}{\left(qt;\;q\right)}_{2k}}{\left(-1\right)}^{k}q^{\frac{k(k-1)}{2}}t^{k}\\ \;=\frac{1}{1\;-\;t}\sum_{k=0}^{\infty }\frac{{\left(q^{\frac{1}{2}};\;q\right)}_{k}{\left(-q^{\frac{1}{2}};\;q\right)}_{k}{\left(-q;\;q\right)}_{k}{\left(q^{\frac{z+m+1}{2}};\;q\right)}_{k}{\left(-1\right)}^{k}q^{\frac{k(k-1)}{2}}}{{\left(q;\;q\right)}_{k}{\left(q^{m+1};\;q\right)}_{k}{\left({\left(qt\right)}^{\frac{1}{2}};\;q\right)}_{k}{\left(-{\left(qt\right)}^{\frac{1}{2}};\;q\right)}_{k}{\left(qt^{\frac{1}{2}};\;q\right)}_{k}{\left(-qt^{\frac{1}{2}};\;q\right)}_{k}}t^{k}, \end{array}$$

which completes the proof. Note that upon comparison with (1), one requires the vanishing numerator element in the basic hypergeometric series due to the factor ${\left(-1\right)}^{k}q^{\frac{k(k-1)}{2}}$ in the sum, so it is not of type _4_*ϕ*_5_. □

Observe that we obtain (12) by setting *m* = 0 in (22).

### Theorem 4.

*Let q*, *t*, *z*, λ∈ℂ, |*q*| < 1, |*t*| < 1. *Then the q-Sylvester polynomials satisfy the following generating functions:*

(23) ∑n=0∞(qλ;q)nφn(z;q)tn=1(zt;q)λϕ21(qλ,qzztqλ|q,t),

(24) $$\sum_{n=0}^{\infty }\varphi_{n}(z;\;q)t^{n}=\frac{1}{{\left(t;\;q\right)}_{z}{\left(zt;\;q\right)}_{\infty }},$$

where the principal values of q^z^ and q^λ^ are taken.

### Proof.

Let us prove the generating function (23) by using an analogous method as before, namely

$$\begin{array}{l} \sum_{n=0}^{\infty }{\left(q^{\lambda };\;q\right)}_{n}\varphi_{n}(z;\;q)t^{n}=\sum_{n=0}^{\infty }\sum_{k=0}^{n}\frac{{\left(q^{-n};\;q\right)}_{k}{\left(q^{z};\;q\right)}_{k}{\left(-1\right)}^{k}q^{-\frac{k(k-1)}{2}}{\left(q^{\lambda };\;q\right)}_{n}}{{\left(q;\;q\right)}_{n}{\left(q;\;q\right)}_{k}}q^{nk}z^{n-k}t^{n}\\ \;=\sum_{n=0}^{\infty }\sum_{k=0}^{n}\frac{{\left(q^{z};\;q\right)}_{k}{\left(q^{\lambda };\;q\right)}_{n}}{{\left(q;\;q\right)}_{n-k}{\left(q;\;q\right)}_{k}}z^{n-k}t^{n}=\sum_{k=0}^{\infty }\sum_{n=0}^{\infty }\frac{{\left(q^{z};\;q\right)}_{k}{\left(q^{\lambda };\;q\right)}_{n+k}}{{\left(q;\;q\right)}_{n}{\left(q;\;q\right)}_{k}}z^{n}t^{n+k}\\ \;=\sum_{k=0}^{\infty }\frac{{\left(q^{z};\;q\right)}_{k}{\left(q^{\lambda };\;q\right)}_{k}}{{\left(q;\;q\right)}_{k}}t^{k}\sum_{n=0}^{\infty }\frac{{\left(q^{\lambda +k};\;q\right)}_{n}{\left(zt\right)}^{n}}{{\left(q;\;q\right)}_{n}}=\sum_{k=0}^{\infty }\frac{{\left(q^{z};\;q\right)}_{k}{\left(q^{\lambda };\;q\right)}_{k}}{{\left(q;\;q\right)}_{k}{\left(zt;\;q\right)}_{\lambda +k}}t^{k}\\ \;=\frac{1}{{\left(zt;\;q\right)}_{\lambda }}\sum_{k=0}^{\infty }\frac{{\left(q^{z};\;q\right)}_{k}{\left(q^{\lambda };\;q\right)}_{k}}{{\left(q;\;q\right)}_{k}{\left(ztq^{\lambda };\;q\right)}_{k}}t^{k}. \end{array}$$

Since |*q*| < 1, (24) follows from taking *λ* → ∞ and applying the *q*-binomial theorem. □

Now we find the *q*-analogue of the first generating function for the *q*-Cesàro polynomials (9).

### Theorem 5.

*Let t*, *z*, s∈ℂ, |*t*| < 1, |*z*| < 1. *Then the q-Cesàro polynomials satisfy the following generating function:*

$$\sum_{n=0}^{\infty }g_{n}^{\left(s\right)}(z;\;q)t^{n}=\frac{1}{\left(1\;-\;tzq^{s}\right){\left(t;\;q\right)}_{s+1}}.$$

### Proof.

Let us prove this by using (11) and some basic properties of the *q*-Pochhammer symbols and the *q*-binomial coefficient. One has

$$\begin{array}{l} \sum_{n=0}^{\infty }g_{n}^{\left(s\right)}(z;\;q)t^{n}=\sum_{n=0}^{\infty }\sum_{k=0}^{n}{\left[\begin{array}{c} k+s\\ s \end{array}\right]}_{q}{\left(zq^{s}\right)}^{n-k}t^{n}=\sum_{n=0}^{\infty }\sum_{k=0}^{n}{\left[\begin{array}{c} n-k+s\\ s \end{array}\right]}_{q}{\left(zq^{s}\right)}^{k}t^{n}\\ \;=\sum_{k=0}^{\infty }{\left(tzq^{s}\right)}^{k}\sum_{n=0}^{\infty }{\left[\begin{array}{c} n+s\\ s \end{array}\right]}_{q}t^{n}=\frac{1}{\left(1\;-\;tzq^{s}\right){\left(t;\;q\right)}_{s+1}}\text{,} \end{array}$$

where we have used the geometric series, (5), and (6), which completes the proof. □

The demonstration that we obtain (9) upon taking the limit *q* ↑ 1 follows by using (5) and then (6).

### Remark 1.

*We were unable to find the q-analogue of the second generating function (*10*) for the q-Cesàro polynomials. The method which Agarwal and Manocha [11] used to obtain (*10*) does not seem to straightforwardly generate a corresponding q-analogue. Furthermore, using (*8*), one can see that (*10*) is equivalent to the following generating function for Jacobi polynomials [12] (3.15)*

(25) $$\sum_{n=0}^{\infty }\left(\begin{array}{c} m+n\\ m \end{array}\right)P_{m+n}^{\left(\alpha ,\beta -n\right)}(x)t^{n}={\left(1-t\right)}^{\beta }{\left(1-\frac{1}{2}(x+1)t\right)}^{-\alpha -\beta -m-1}P_{m}^{\left(\alpha ,\beta \right)}\left(\frac{x\;-\;\frac{1}{2}(x\;+\;1)t}{1\;-\;\frac{1}{2}(x\;+\;1)t}\right).$$

*Unfortunately, this formula does not seem amenable to a natural q-analogue. Note that* (25) *is given with a misprint in [6] (p. 165, Problem 9(ii)).*

### Remark 2.

*It has been mentioned by a referee that* Theorems 4, 5 *can be derived from the results contained in [13]. However, it is not clear to the authors how to go from the q-Bernoulli polynomials to the q-Sylvester and q-Cesàro polynomials. Moreover, the generating functions for q-Sylvester and q-Cesàro polynomials do not look similar to the generating functions given in [13].*

## 5. Conclusions

In this paper, we introduced several *q*-polynomials and derived *q*-analogues of most of the known generating functions for these polynomials. In particular, this was accomplished for the Bateman, Sylvester, Pasternack, and Cesàro polynomials. In Corollary 1, we also were able to find new classical generating functions, by taking *q* ↑ 1 limits of the *q*-generating functions we obtained. We were unable to find a *q*-analogue for the classical generating function for *q*-Cesàro polynomials (10) (see Remark 1). This would be an interesting project for the future. It would be interesting to see if it is possible to use *q*-calculus to obtain *q*-analogues of the results obtained in [3].

### Remark 3.

*Note that we recently discovered that the Ph.D. thesis of Mohammad Asif [14] (Chapter 4), under the direction of Prof. Mumtaz Ahmad Khan, contains some of the material that appears in this manuscript. Asif treats both q-Bateman polynomials, the q-Pasternack polynomials, and the q-Cesàro polynomials, all of which are defined in precisely the same way, although Asif uses different notations to display these polynomials. Asif also treats q-Shively pseudo–Laguerre polynomials and q-Gottlieb polynomials. Asif does not treat the q-Sylvester polynomials. It should be noted, however, that Asif arrives at the wrong conclusions for the lower parameters in* Theorem 1 *and in* (17). *His notation may or may not be at fault in his representation of* Theorem 5. *He does find the correct result for* Theorem 3.
